# Carbohydrate Hydrolase-Inhibitory Activity of Juice-Based Phenolic Extracts in Correlation to Their Anthocyanin/Copigment Profile

**DOI:** 10.3390/molecules25225224

**Published:** 2020-11-10

**Authors:** Kirsten Berger, Johanna Josefine Ostberg-Potthoff, Tamara Bakuradze, Peter Winterhalter, Elke Richling

**Affiliations:** 1 Department of Chemistry, Division of Food Chemistry and Toxicology, Technische Universität Kaiserslautern, Erwin-Schrödinger-Str. 52, 67663 Kaiserslautern, Germany; berger@chemie.uni-kl.de (K.B.); bakuradze@chemie.uni-kl.de (T.B.); 2 Institut für Lebensmittelchemie, Technische Universität Braunschweig, Schleinitzstr. 20, D-38106 Braunschweig, Germany; j.ostberg@tu-bs.de (J.J.O.-P.); p.winterhalter@tu-bs.de (P.W.)

**Keywords:** α-amylase, α-glucosidase, inhibition, red fruits, anthocyanins, phenolic compounds, blood glucose

## Abstract

Red fruits and their juices are rich sources of polyphenols, especially anthocyanins. Some studies have shown that such polyphenols can inhibit enzymes of the carbohydrate metabolism, such as α-amylase and α-glucosidase, that indirectly regulate blood sugar levels. The presented study examined the in vitro inhibitory activity against α-amylase and α-glucosidase of various phenolic extracts prepared from direct juices, concentrates, and purees of nine different berries which differ in their anthocyanin and copigment profile. Generally, the extracts with the highest phenolic content—aronia (67.7 ± 3.2 g GAE/100 g; cyanidin 3-galactoside; chlorogenic acid), pomegranate (65.7 ± 7.9 g GAE/100 g; cyanidin 3,5-diglucoside; punicalin), and red grape (59.6 ± 2.5 g GAE/100 g; malvidin 3-glucoside; quercetin 3-glucuronide)—showed also one of the highest inhibitory activities against α-amylase (326.9 ± 75.8 μg/mL; 789.7 ± 220.9 μg/mL; 646.1 ± 81.8 μg/mL) and α-glucosidase (115.6 ± 32.5 μg/mL; 127.8 ± 20.1 μg/mL; 160.6 ± 68.4 μg/mL) and, partially, were even more potent inhibitors than acarbose (441 ± 30 μg/mL; 1439 ± 85 μg/mL). Additionally, the investigation of single anthocyanins and glycosylated flavonoids demonstrated a structure- and size-dependent inhibitory activity. In the future in vivo studies are envisaged.

## 1. Introduction

Small fruits and their juices are rich sources of polyphenols, especially anthocyanins. The anthocyanins are a sub-group of flavonoids that primarily occur in violet, blue, and red fruits. They are water-soluble plant pigments and serve as an attractant, as well as a protector against oxidative stress and excessive light. Their hue is determined by the pH and the presence of copigments [1]. Copigments or co-factors are colorless flavonoids and phenolic acids which form a non-covalently linked complex with anthocyanins [2]. Flavonoids, especially anthocyanins, have been studied extensively over the last few decades because of their diverse beneficial health effects; inter alia they exhibit antioxidant, antibacterial, antiviral, anti-inflammatory, and antidiabetic activity [1,2,3,4,5,6,7].

Diabetes mellitus is a major global health problem: in 2019 463 million people suffered from diabetes worldwide, and its global prevalence is increasing. It is commonly caused by impaired insulin secretion, with or without insulin resistance, and is characterized by hyperglycemia [8]. There are two predominant types. Type I diabetes is caused by a lack of insulin as a result of destroyed β-cells, while type 2 diabetes is the result of insulin resistance [8].

The inhibition of carbohydrate digestive enzymes such as α-amylase and α-glucosidase is one of the therapeutic strategies to control postprandial hyperglycemia and also the basis of the antidiabetic effects of some plant foods [9]. The digestion and absorption of carbohydrates involves the hydrolysis of complex polysaccharides into absorbable monosaccharides. Key enzymes in this process are α-amylases (EC 3.2.1.1), such as salivary and pancreatic amylase. α-Amylases catalyze the hydrolysis of starch into smaller fragments including disaccharides (maltose). The further hydrolysis of these disaccharides is catalyzed by α-glucosidase (EC 3.2.1.20), a specific membrane-bound enzyme in the small intestine [10].

Some inhibitors of these digestive enzymes have been approved for use as medicines to treat type I and II diabetes, namely acarbose, miglitol, and voglibose [11]. In addition, some polyphenols reportedly exhibit similar inhibitory activity. In particular, flavonoids showed some potential in the past. Flavonoid molecules have several hydroxyl groups, allowing them to strongly bind to and inhibit the catalytic centers of carbohydrate digestion enzymes [12]. However, not only does the number of hydroxyl groups influence the inhibitory potential, but the position of those groups also plays an important role. The hydroxylation of the C ring, for example, could weaken the binding at the digestive enzymes [13]. Besides this, van der Waals forces play an important role for the inhibition potential of a substance [14]. A wide range of polyphenols reportedly inhibit α-amylase, whereas α-glucosidase appears to be primarily sensitive to anthocyanins [15]. This inhibitory potential has been verified by studying polyphenol-containing extracts from various fruits, including blueberries, pomegranates, and strawberries [9,10,15]. With the help of activity-guided fractionation numerous potent inhibitors in berries could already been identified. This includes besides anthocyanins mainly flavonoids, and ellagitannins. In this context, a prevalence for inhibition of the α-glucosidase has also been examined [16].

This study focused on extracts prepared from red fruit juices. The extracts were tested in an enzyme inhibition assay to determine their inhibitory activity against α-amylase and α-glucosidase. Additionally, isolated anthocyanins and copigments were investigated to create a link between the molecular structure and the inhibitory potential of individual phenolic compounds and also to examine possible synergistic and antagonistic mechanism.

## 2. Results and Discussion

Thirty-eight fruit products derived from aronia, bilberry, blackcurrant, cranberry, elderberry, lingonberry, pomegranate, red grape, and sour cherry were extracted and investigated with regard to their inhibition of α-glucosidase and α-amylase activity, including direct juices (not from concentrate, NFC), concentrates (juice concentrate, JC), and purees.

### 2.1. Phenolic Composition of the Extracts

In a recently published article, we presented the phenolic composition of different extracts in detail [16]. Here, we focus on the major anthocyanins and copigments of all nine fruit varieties (see Table 1). The discussion in this section focuses on the extracts that yielded the most notable results. The aronia juice products were richest in proanthocyanidins, anthocyanins, and phenolic acids. Most of these compounds were cyanidin derivatives, including two derivatives (namely cyanidin-3,5-hexoside-(epi)catechin and cyanidin-3-pentoside-(epi)catechin) that were first identified by Oszmiański and Lachowicz [17]. The pomegranate juice extracts were particularly rich in hydrolyzable tannins and diglycosidically-bound anthocyanins but contained only small quantities of anthocyanins in general. A wide spectrum of anthocyanin glucosides was identified in the extracts from red grape juice, including delphinidin-, cyanidin-, petunidin-, peonidin-, and malvidin-glucosides. These extracts were also rich in flavonols and proanthocyanidins.

**Figure 1:**
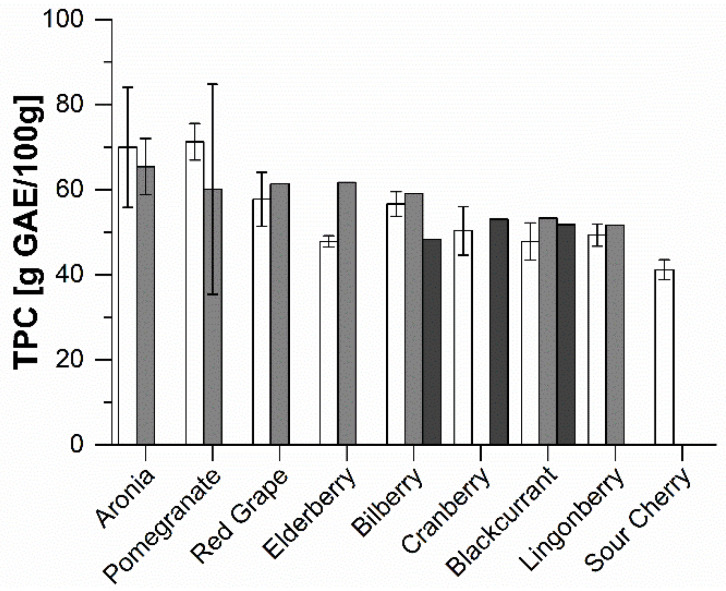
Total phenolic contents (TPC) of the extracts. Results are shown for extracts of direct juice (white), juice concentrate (light grey), and purees (dark grey), and are presented as means ± SD (*n* = 3). GAE = gallic acid equivalent.

The total phenolic contents (TPC) of the extracts are presented in Figure 1. Pomegranate, aronia, and red grape products had the highest TPC values, while sour cherry products had the lowest. Concentrates generally had higher TPCs than juices. The lower phenolic content of the purees was attributed to the sample preparation which included a pre-extraction step (for details see Section 3.2).

The extracts with the highest total phenolic contents determined by the Folin-Ciocalteu method were those derived from pomegranate, aronia, and red grape. In these three extracts, the composition of polyphenols determined by HPLC-MS analysis was quite different. Aronia juice extracts contained mostly anthocyanins and procyanidins, as reported previously [17]. Pomegranate juice extracts are characterized by lower levels of anthocyanins and high amounts of hydrolyzable tannins [18]. All of the examined red grape juice extracts contained anthocyanins, flavonols, and proanthocyanidins, which is again consistent with previous reports indicating that these fruits contain a wide spectrum of anthocyanins, phenolic acids, flavones, and flavanols [19].

### 2.2. In Vitro Inhibition Study

**Figure 2:**
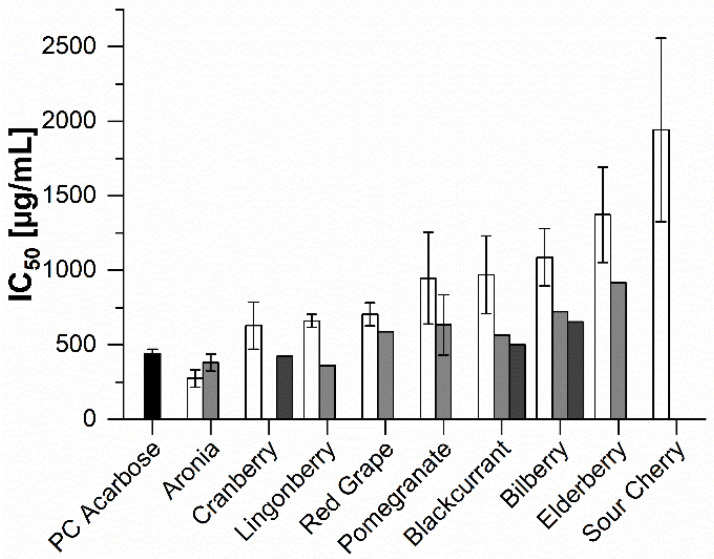
Inhibitory activities (IC_50_) of the red fruit product extracts against α-amylase. Results are shown for extracts of direct juice (white), juice concentrate (light grey), purees (dark grey), and the positive control (PC) acarbose. Bars represent means ± SD (*n* = 3).

The pseudotetrasaccharide acarbose, which is a well-known α-amylase inhibitor marketed under the brand name Glucobay^®^, was used as the positive control (PC); its IC_50_ value (half-inhibitory concentration) was determined to be 441 ± 30 μg/mL. Figure 2 shows the mean IC_50_ values for the fruit product extracts (amounts of samples: 1–4). All of the aronia products (direct juice IC_50_ = 273 ± 57 μg/mL; concentrate IC_50_ = 381 ± 57 μg/mL) had lower IC_50_ values than the positive control, as had the extracts prepared from lingonberry concentrate (IC_50_ = 361 μg/mL) and cranberry puree (IC_50_ = 424 μg/mL). These products were the strongest inhibitors among the studied red fruit extracts. In general, the extracts of the juice concentrates exhibited stronger inhibitory activity than those of the direct juices. However, extracts of aronia direct juice (273 ± 57 μg/mL) were marginal more potent inhibitors than the juice concentrate extracts (381 ± 57 μg/mL). In the α-amylase inhibition screening, all of the puree extracts were more active than the corresponding direct juice extracts, although only purees of cranberry, blackcurrant, and bilberry were investigated (IC_50_ = 424, 501, and 655 μg/mL, respectively). The direct juice extracts of sour cherry (IC_50_ = 1943 ± 615 μg/mL), elderberry (IC_50_ = 1373 ± 320 μg/mL), and bilberry (IC_50_ = 1088 ± 192 μg/mL) were the weakest α-amylase inhibitors among the studied products. In this assay there were no significant differences observed between the investigated red grapes varieties, except for one sample juice concentrate from Italian grapes which demonstrated half the inhibitory activity than the others.

**Figure 3:**
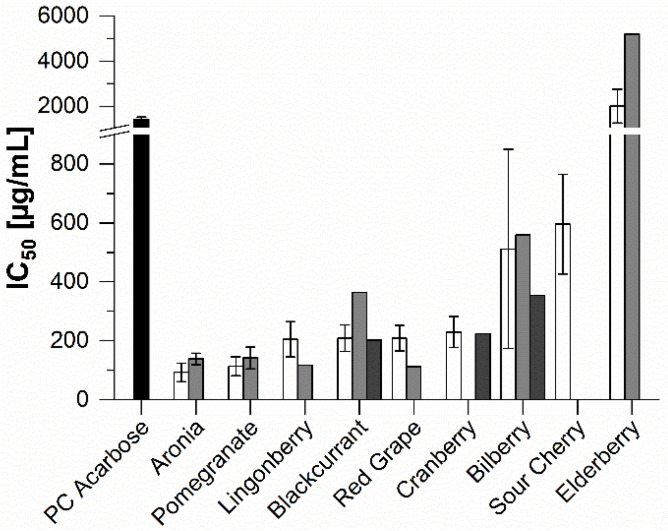
Inhibitory activities (IC_50_) of the red fruit product extracts against α-glucosidase. Results are shown for extracts of direct juice (white), juice concentrate (light grey), purees (dark grey), and the positive control (PC) acarbose. Bars represent means ± SD (*n* = 3)

The IC_50_ values of the red juice extracts used in the α-glucosidase assay are shown in Figure 3. The α-glucosidase inhibitor acarbose (IC_50_ = 1439 ± 85 μg/mL) was used as the positive control (PC). All the studied extracts other than those derived from elderberries (direct juice IC_50_ = 2014 ± 743 μg/mL; concentrate IC_50_ = 5201 μg/mL) had lower IC_50_ values than the positive control and thus had higher inhibitory activities. The puree extracts (blackcurrant: IC_50_ = 203 μg/mL; cranberry: IC_50_ = 224 μg/mL; bilberry: IC_50_ = 355 μg/mL) had very similar inhibition potentials to the corresponding direct juice extracts, whose IC_50_ values were 208 ± 44 μg/mL, 230 ± 53 μg/mL and 512 ± 337 μg/mL, respectively. Interestingly, although the juice concentrate extracts were generally stronger inhibitors of α-amylase than the corresponding direct juices, the reverse was true for α-glucosidase inhibition. The only exceptions were lingonberry and red grape, whose concentrates had mean IC_50_ values of 118 μg/mL and 112 μg/mL, respectively, whereas the direct juices had IC_50_ values of 205 ± 59 μg/mL and 209 ± 43 μg/mL, respectively. Aside from these two juice concentrate extracts, the NFC extracts from aronia and pomegranate, which had IC_50_ values of 93 ± 31 μg/mL and 113 ± 32 μg/mL, were the most active amongst the studied fruit products. The examined red grape varieties showed no difference in their inhibitory potential.

Both, the α-amylase and α-glucosidase inhibition assay showed that the extracts made from purees were the most potent inhibitors for each fruit type. The increased inhibitory activity compared to those of direct juice extracts was reflected in higher phenolic contents. In the α-amylase inhibition assay, the extracts from concentrates were generally more potent than the corresponding direct (NFC) juices; except for aronia. Controversially, in the α-glucosidase inhibition assay, extracts from concentrates were generally less active than those from direct juices; the only fruits for which this trend did not hold were lingonberry and bilberry.

Extracts of sour cherry, elderberry, and bilberry exhibited comparatively weak α-amylase inhibition, whereas those of aronia, lingonberry, cranberry, and red grape were more potent. The strongest α-glucosidase inhibitors were extracts from aronia, pomegranate, lingonberry, and red grape, whereas extracts of bilberry, sour cherry, and elderberry were less active. This is consistent with literature reports indicating that aronia extracts showed higher α-glucosidase and α-amylase inhibitory activity than extracts of other red fruits such as sour cherry [20]. In both assays the different investigated red grape varieties showed related inhibitory activities. Therefore, it seems that the variety does not influence the potential to inhibit α-amylase and α-glucosidase. Overall, extracts prepared from red fruit juices showed higher inhibitory activity towards α-glucosidase and some even towards α-amylase than the antidiabetic compound acarbose. Our results are in line with literature data in which for acarbose a wide range of IC_50_ values has already been described. It varied from 50 μg/mL to 10 mg/mL for α-amylase [21]. The high inhibitory activities of aronia and pomegranate extracts were mirrored by high total phenolic contents, which were around twice as high as those of the sour cherry extracts. However, lingonberry and cranberry extracts showed high inhibitory activities against the two digestive enzymes while having low TPC values and extracts of elderberry and bilberry had weak inhibitory activities but high TPC values. This suggests that inhibitory activity is not only dependent on the total content of polyphenols (i.e., the TPC value) but also on the presence of specific subgroups of polyphenols [22].

### 2.3. Investigation of Single Anthocyanins and Copigments

**Figure 4:**
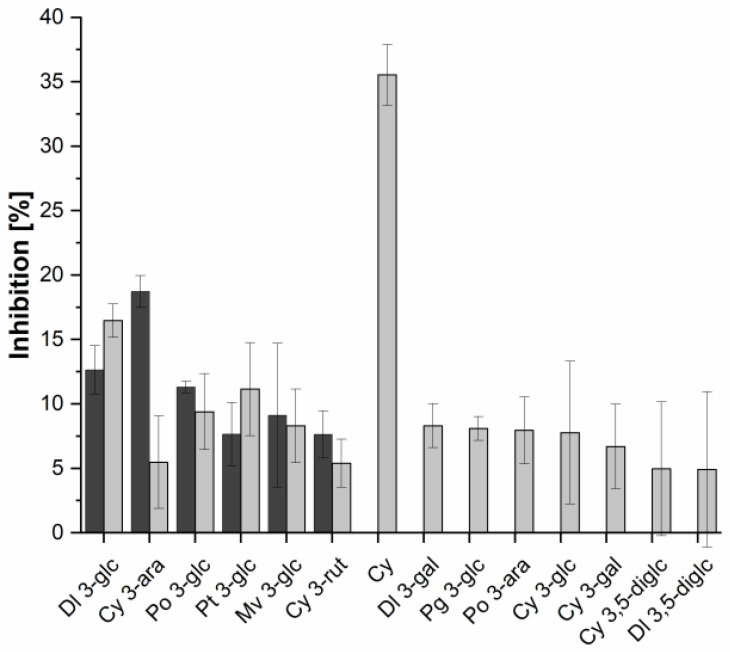
Inhibition (%) of α-amylase (dark grey) and α-glucosidase (light grey) assay. Results are shown for anthocyanin standards. Bars represent means ± SD (*n* = 3). Cy = Cyanidin, Mv = Malvidin, Pt = Petunidin, Po = Peonidin, Dl = Delphinidin, Pg = Pelargonidin, rut = rutinoside, glc = glucoside, ara = arabinoside, diglc = diglucoside, gal = galactoside.

To identify the active ingredients in the extracts as well as to investigate possible synergistic effects, different commercially available standards from anthocyanins and copigments were examined. For all tested samples the IC_50_ value was higher than 2 mM. Therefore, the % inhibition of 2 mM of each single substance was compared as shown in Figure 4. In general, anthocyanins showed a higher inhibitory potential for α-glucosidase. Cyanidin exhibited with an inhibition of 35.5 ± 2.4% the highest potential for α-glucosidase inhibition. Among the cyanidin derivatives, cyanidin 3-glucoside with 7.8 ± 5.5% inhibition was the next active substance while cyanidin 3,5-diglucoside (inhibition = 4.9 ± 5.2%) was the less active compound. In the α-amylase study, cyanidin 3-arabinoside showed with an inhibition of 18.7 ± 1.2% the highest inhibitory potential followed by the glucosides of delphinidin, peonidin, malvidin, and petunidin (inhibition: 12.7 ± 1.9%; 11.3 ± 0.5%; 9.1 ± 5.6%; 7.7 ± 2.5%) as well as cyanidin 3-rutinoside with 7.6 ± 1.8% inhibition. For a total of eight substances no inhibition of α-amylase activity could be determined. By trend, glycosides (mainly glucosides) of other aglycons than cyanidin (delphinidin, peonidin, petunidin, and malvidin) showed higher inhibitory activities in both inhibition studies. Furthermore, substances with a smaller sugar group attached inhibited the digestive enzymes more effectively.

**Figure 5:**
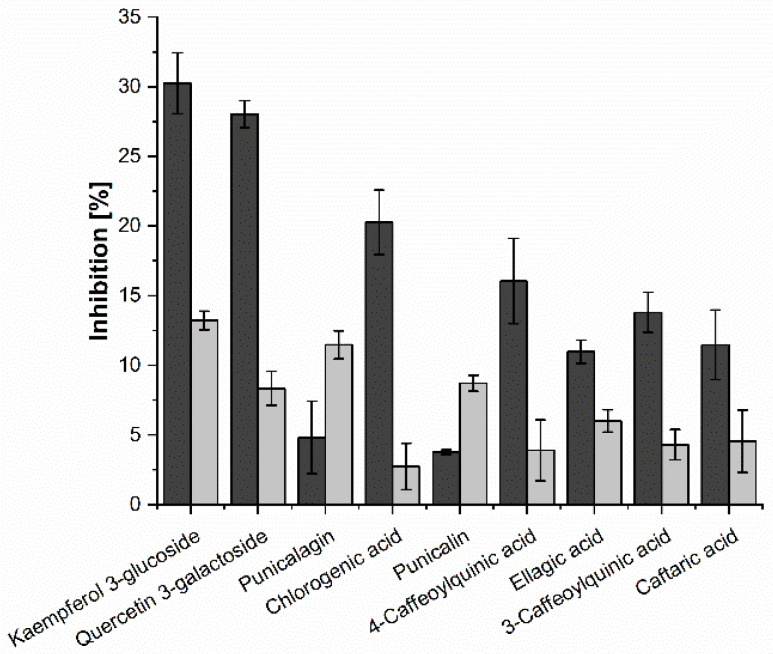
Inhibition (%) of α-amylase (dark grey) and α-glucosidase (light grey) assay. Results are shown for copigment standards. Bars represent means ± SD (*n* = 3).

The results of the commercially available copigment standards tested in both assays are shown in Figure 5. The tested substances in concentrations of 2 mM each were generally more potent inhibitors of α-amylase than α-glucosidase except for punicalagin and punicalin. The most active copigment in both assays was kaempferol 3-glucoside (inhibition: 30.3 ± 2.2% for α-amylase; 13.2 ± 0.7% for α-glucosidase), followed by quercetin 3-galactoside with inhibition values of 28.0 ± 1.0% and 8.3 ± 1.2% for α-amylase and α-glucosidase, respectively. On the other side, caftaric acid was not a very potent inhibitor of the two digestive enzymes (11.5 ± 2.5% inhibition for α-amylase; 4.6 ± 2.2% inhibition for α-glucosidase). Ellagic acid, punicalin, and punicalagin, as well as chlorogenic acid, 3-caffeoylquinic acid and 4-caffeoylquinic acid showed comparable inhibitory activities in both assays, which could be due to their structural similarity. While punicalin (3.8 ± 0.2% inhibition) and punicalagin (4.8 ± 2.6% inhibition) as well as the two caffeoylquinic acids (inhibition: 4-caffeoylquinic acid: 16.0 ± 3.1%; 3-caffeoylquinic acid: 13.8 ± 1.4%) demonstrated a slightly lower inhibition of α-amylase activity than ellagic acid (11.0 ± 0.8% inhibition) and chlorogenic acid (20.3 ± 2.3% inhibition), a contrary observation could be made for α-glucosidase (inhibition: ellagic acid: 6.0 ± 0.8%; punicalin: 8.7 ± 0.6%; punicalagin: 11.5 ± 1.0%; chlorogenic acid: 1.6 ± 3.1%; 4-caffeoylquinic acid: 4.5 ± 3.3%; 3-caffeoylquinic acid: 3.6 ± 2.2%).

Generally, very low inhibition (%) could be determined for all investigated anthocyanins and copigments for both enzymes. For α-amylase there was even no inhibitory activity measurable for some anthocyanins. The investigation of the anthocyanins showed a certain structure dependency of the inhibition potential. With more hydroxyl groups being present in a molecule, higher inhibitory activity was observed. The same tendency was found for the copigments. This is because the hydroxyl groups are able to strongly bind to the catalytic center and following from this inhibit this center [12,23,24]. Besides this, the position of the hydroxylation, and also methylation, plays an important role. The lack of methylation of the B ring of anthocyanins, for example, leads to a stronger binding potential and in consequence to a higher inhibitory activity [13]. An example is delphinidin, which showed a relatively high inhibitory potential with exception for delphinidin 3,5-diglucoside. This is a hint that anthocyanins with a smaller sugar moiety inhibited the two digestive enzymes more efficiently. These results are in line with the investigation that the sugar units which are linked to anthocyanins influence the inhibitory potential, which has already been described for different cyanidin glycosides [25]. This size dependence was also examined for the copigments. Here, punicalin and other ellagitannins inhibited α-amylase and α-glucosidase activity rather weak despite their high amount of hydroxyl groups. Generally, by trend it could be observed that the lower the molecular weight of a substance the higher their inhibitory potential, which also corresponds with literature data [26]. Van der Waals forces influence the inhibitory activity against α-glucosidase [14]. Cyanidin showed the highest % inhibition for α-glucosidase in the presented study and it has been reported before that this anthocyanin can form quit high van der Waals forces. Due to the fact, that for some anthocyanins no inhibition could be determined for α-amylase, a preference for α-glucosidase cannot be denied. This holds true with investigations already described in literature [15]. Altogether, the inhibitory potential of the anthocyanin and copigment standards did not reflect the results of the fruit juice extracts in total. Therefore, it is not possible to trace the high inhibitory potential of the fruit extracts back to one single compound. This suggests that the inhibition also depends on the interaction of different phenolic substances. This suggestion has already been described before [27].

## 3. Materials and Methods

### 3.1. Chemicals

2-Chloro-4-nitrophenyl-α-d-maltotrioside (CNPG3), 4-nitrophenyl-α-d-glucopyranoside (pNGP), α-amylase from porcine pancreas, α-glucosidase from Saccharomyces cerevisiae, ethanol (≥ 99.8%) and Amberlite^®^ XAD-7 were purchased from Sigma-Aldrich (Taufkirchen, Germany). Acarbose was obtained as Glucobay^®^ (50 mg acarbose/tablet) from Bayer Pharmaceuticals (Leverkusen, Germany). Gallic acid monohydrate and sodium carbonate were purchased from Fluka (Buchs, Switzerland). The Folin-Ciocalteu reagent was obtained from Merck (Darmstadt, Germany). Anthocyanin and copigment standards were obtained from Phytolab (Vestenbergsgreuth, Germany) and Extrasynthèse (Genay Cedex, France).

### 3.2. Materials and Preparation of Extracts

Products derived from juices and juice products from nine different red fruits were screened, including aronia (Aronia melanocarpa), bilberry (Vaccinium myrtillus L.), blackcurrant (Ribes nigrum L.), cranberry (Vaccinium macrocarpon), elderberry (Sambucus nigra L.), lingonberry (Vaccinium vitis-idaea L.), pomegranate (Punica granatum L.), red grape (Vitis vinifera L.), and sour cherry (Prunus cerasus L.). As different varieties of red grapes are marketed, we investigated the inhibitory potential of varieties such as Vitis labrusca “Concord” and Vitis vinifera “Lambrusco” as well as samples and extracts originating from French, Spanish, and Italian grapes, respectively. In total, the study investigated twenty-six samples of direct juices, nine from concentrates, and three from purees.

The puree samples were subjected to batch extraction with ethanol/water 19:1 *v*/*v* (stirring overnight at room temperature). After filtration, solvent was evaporated under reduced pressure at 40 °C and the samples were lyophilized.

Each of the puree samples, direct juices and juice concentrates were applied onto an Amberlite^®^ XAD-7 column. The column was washed with water in order to eliminate carbohydrates, proteins and minerals. The phenolic compounds were then eluted with a mixture of ethanol/water 19:1 *v*/*v*. Solvents were evaporated under reduced pressure at 40 °C and the XAD-7 extracts were freeze-dried.

### 3.3. HPLC/DAD/ESI-MS^n^ Analysis

The HPLC system (1100/1200 series, Agilent, Waldbronn, Germany) consisted of a binary pump (G1312A), an autosampler (G1329B), and a DAD-detector (G1316A). It was coupled to a HCT Ultra Ion Trap mass spectrometer (Bruker Daltonics, Bremen, Germany) with an electrospray ionization source (ESI). The anthocyanins and copigments were separated on a Luna C18(2) 3 μm column (150 × 2.0 mm, Phenomenex, Aschaffenburg, Germany) using water/acetonitrile/formic acid 95:3:2 *v*/*v*/*v* (eluent A) and water/acetonitrile/formic acid 48:50:2 *v*/*v*/*v* (eluent B) at a flow rate of 200 μL/min. Gradient elution was performed, starting with 6% eluent B and rising to 35% over 30 min. The level of eluent B was then set to 40% until minute 35 and then 90% until 45 min. This level was maintained for 5 min before being reduced to 30% until 55 min. Finally, the initial conditions (6% eluent B) were restored until 70 min. The ESI source was operated in positive mode (anthocyanins), negative mode (copigments) and alternating mode (+/− 3000 V), using nitrogen as the nebulizer gas (60 psi) and the drying gas (11 L/min, 330 °C). The sample extracts were dissolved in 2 mL eluent A. An aliquot of 5 μL was analyzed by the HPLC/DAD/ESI-MS^n^ method described above using the Bruker Hystar V.3.2, Bruker ESI-Compass 1.3 for HCT/Esquire, and Data Analysis Version 3.0 software packages (Bruker Daltonics, Bremen, Germany).

### 3.4. Folin-Ciocalteu Assay

The total phenolic content (TPC) of the samples was determined using the Folin-Ciocalteu method as described previously with slight modifications [28]. Briefly, 200 μL aliquots of the sample, a standard, and a blank solution were each mixed with 1 mL Folin-Ciocalteu reagent (diluted 1:10 from stock solution) in a semi-microcuvette. Between 30 s and 8 min later, 800 μL 7.5% Na_2_CO_3_ solution was added at the same time to each mixture and incubated for 2 h at room temperature. The extinction was then measured at λ 760 nm using a spectrophotometer (Jasco, Groß-Umstadt, Germany). The TPC was calculated with a calibration curve constructed using gallic acid solutions. Each sample was measured in triplicates, and the results were expressed as grams of gallic acid equivalents per 100 g extract (g GAE/100 g).

### 3.5. In Vitro α-Amylase Inhibition Study

The α-amylase inhibition assay was based on a previously described spectrophotometric method [29]. Acarbose was used as the reference compound. Briefly, 20 μL aliquots of the sample (in five different concentrations), the positive control, or the negative control were each mixed with 70 μL of 40 mM phosphate buffered saline (PBS) at pH 6.9 containing 30 U/mL of porcine pancreatic α-amylase and incubated in a 96-well microplate at 37 °C for 10 min. After pre-incubation, 100 μL of substrate solution (40 mM PBS with 4 mM 2-chloro-4-nitrophenyl-α-d-maltotrioside) was added, and the resulting mixtures were incubated at 37 °C for 8 min. Their absorbance was then measured at λ 405 nm using a microplate reader (Biotek, Bad Friedrichshall, Germany). Each sample was measured in triplicate.

### 3.6. In Vitro α-Glucosidase Inhibition Study

The α-glucosidase inhibition assay used 4-nitrophenyl-α-d-glucopyranoside as substrate. 20 μL aliquots of the sample (in five different concentrations) or negative and positive control solutions were each mixed with 70 μL enzyme solution (1 U/mL) in 0.1 M PBS (pH 6.8). After incubation at 25 °C for 10 min, 100 μL of 4 mM pNPG solution in PBS was added and incubated in a 96-well microplate for 5 min at 25 °C. The absorbance was measured at λ 405 nm using a microplate reader (Biotek, Bad Friedrichshall, Germany). Each sample was measured in triplicate.

## 4. Conclusions

It has been reported that the inhibition of α-glucosidase activity by fruit products is mainly due to anthocyanins [15]. Our results are consistent with this finding: extracts from anthocyanin-rich red fruit juices and juice products were generally stronger inhibitors of α-glucosidase than α-amylase. The investigation of single compounds pointed out that potential synergistic effects should be taken into account. Therefore, further studies using fractionated extracts will be needed to identify the single polyphenols or groups of polyphenols being responsible for inhibiting the activity of the two digestive enzymes α-glucosidase and α-amylase. Consequently, as a next step, human intervention studies have to be performed to verify our observations.

## Figures and Tables

**Table 1:** Selection of anthocyanins and copigments of the nine red fruits included in the study.

Compound	Mode [M + H]^+/−^	Ion *m*/*z*	Fragments *m*/*z*	Red Fruit								
				Ar	Bi	Bc	Cb	Eb	Lb	Pg	Rg	Sc
**Anthocyanin**												
Cyanidin 3-arabinoside	+	419	287	×	×		×		×			
Cyanidin 3,5-diglucoside	+	611	449/287							×		
Cyanidin 3-galactoside	+	449	287	×	×		×		×			
Cyanidin 3-glucoside	+	449	287	×	×	×		×	×	×	×	
Cyanidin 3-(2^G^-glucosyl-rutinoside)	+	757	677/287									×
Cyanidin 3-rutinoside	+	595	287			×						×
Cyanidin 3-sambubioside	+	581	287					×				
Delphinidin 3,5-diglucoside	+	627	303							×		
Delphinidin 3-galactoside	+	465	303		×							
Delphinidin 3-glucoside	+	465	303		×	×				×	×	
Delphinidin 3-rutinoside	+	611	303			×						
Malvidin 3-glucoside	+	493	331		×						×	
Myricetin 3-glucoside	-	479	316			×						
Peonidin 3-arabinoside	+	433	301				×					
Peonidin 3-galactoside	+	463	301				×					
Peonidin 3-glucoside	+	463	301		×		×				×	
Petunidin 3-glucoside	+	479	317		×						×	
**Copigment**												
Caftaric acid	-	311	179								×	
3-Caffeoylquinic acid	-	353	191/179/135	×				×				×
4-Caffeoylquinic acid	-	353	191/179/173	×	×			×				
Chlorogenic acid	-	353	191/179/161	×	×		×	×	×			×
Coumaroyl iridoid	-	535	371		×							
Ellagic acid	-	301	229							×		
Kaempferol 3-glucoside	-	447	285								×	
Punicalagin	-	1083	601							×		
Punicalin	-	781	601							×		
Quercetin 3-galactoside	-	463	301	×	×		×		×			
Quercetin-3-glucoronide	-	477	301								×	
Quercetin 3-rhamnoside	-	447	301						×			
Quercetin 3-rutinoside	-	609	301					×				

Ar = Aronia, Bi = Bilberry, Bc = Blackcurrant, Cb = Cranberry, Eb = Elderberry, Lb = Lingonberry, Pg = Pomegranate, Rg = Red grape, Sc = Sour cherry.

## Abbreviations

GAE	gallic acid equivalents
IC_50_	half maximal inhibitory concentration
JC	juice concentrate
NFC	juice not from concentrate
PC	positive control
TPC	total phenolic content
